# Can Geographical Factors Determine the Choices of Farmers in the Ethiopian Highlands to Trade in Livestock Markets?

**DOI:** 10.1371/journal.pone.0030710

**Published:** 2012-02-15

**Authors:** Angel Ortiz-Pelaez, Getaneh Ashenafi, Francois Roger, Agnes Waret-Szkuta

**Affiliations:** 1 Pinner, Middlesex, United Kingdom; 2 National Veterinary Institute (NVI), Debre Zeit, Ethiopia; 3 AGIRs Unit, CIRAD, Montpellier, France; 4 Veterinary Epidemiology and Public Health, Royal Veterinary College, Hawskhead, Hertfordshire, United Kingdom; Universidad Veracruzana, Mexico

## Abstract

Proximity and affiliation to the local market appear to be two of the most relevant factors to explain farmer's choices to select a particular trading point. Physical barriers may limit the options , especially in developing countries. A network of villages linked by traders/farmer-traders sharing livestock markets was built with field data collected in 75 villages from 8 *kebelles* in the Wassona Werna wereda of the Ethiopian Highlands. Two exponential random graph models were fitted with various geographical and demographic attributes of the nodes (dyadic independent model) and three internal network structures (dyadic dependent model). Several diagnostic methods were applied to assess the goodness of fit of the models. The odds of an edge where the distance to the main market Debre Behran and the difference in altitude between two connected villages are both large increases significantly so that villages far away from the main market and at different altitude are more likely to be linked in the network than randomly. The odds of forming an edge between two villages in Abamote or Gudoberet *kebelles* are approximately 75% lower than an edge between villages in any other *kebelles* (p<0.05). The conditional log-odds of two villages forming a tie that is not included in a triangle, a 2-star or a 3-star is extremely low, increasing the odds significantly (p<0.05) each time a node is in a 2-star structure and decreasing it when a node is in a 3-star (p<0.05) or in a triangle formation (p<0.05)), conditional on the rest of the network. Two major constraining factors, namely distance and altitude, are not deterrent for the potential contact of susceptible small ruminant populations in the Highlands of Ethiopia.

## Introduction

Livestock trade is an activity often occurring via an intermediate step in the form of a market or trading point with various levels of organization, procedures and control. An essential actor in this supply chain is the trader or middleman that represents a conduit between production sites and trading and consumption areas. For disease surveillance and control purposes, the interest lies in the different forms of interaction between production sites where susceptible animals are kept. This interaction usually occurs via a physical medium (fomites) and the actor/s (farmer, trader) for they can act as mechanical carrier of the virus, or move infected animals from an infected to susceptible farms. Markets have been shown to play a role in the dispersal of infectious diseases between livestock premises in countries with intensive and highly technified farming systems [1]–[2]–[3]–[4] although there is not much evidence of the drivers that take farmers to select a particular trading point.

Proximity and affiliation to the local market as part of the community network appear to be two of the most relevant factors to explain farmer's choices. Other factors like biosecurity, animal welfare and environmental compliance are not a priority for both farmers at the time to move livestock to markets and market operators as part of their business operations [5]. This risk-prone behaviour must be interpreted as the negative effect of the attempt to maximize the profitability of the farming enterprise. In developing countries the choices might be conditioned by market demands which usually operate initially at local level and the limited available resources in terms of transportation facilities. *A priori* it seems there is not much difference in the incentives that farmers from different farming systems are presented with to discriminate between multiple trading points.

There has been a recent surge of research efforts to understand the pattern of animals movements and the role of livestock markets in developing countries, mainly due to their putative association with the spread and persistence of infectious diseases like H5N1 Highly Pathogenic Avian Influenza (HPAI) [6]–[7], FMD [8] and Trypanosomiasis [9], to name a few.

The network paradigm allows the integration of such interaction by joining actors represented by farms, production areas or animals and the trader or the trading point represented by the market. This phenomenon can be seen as a 2-mode or affiliation network [10] where the nodes are made of two distinctive classes: a set of actors (villages in our study) and a set of events (markets, traders); and the edges between nodes of different class that represent the choices of the farmers and/or traders to trade their small ruminants in a particular market or trading point. These are the basis of a bipartite graph that can be analyzed itself or transformed into other network structures.

**Figure 1:**
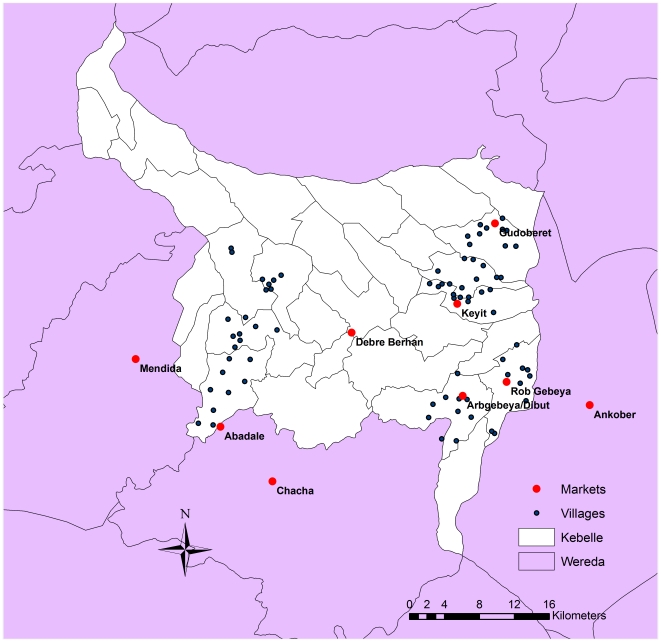
Location of the 75 villages and 9 markets in the Bassona Werna *wereda* region.

Ethiopia has one of the largest population of small ruminants in Africa with 25 million of sheep and 23 million of goats in 2008 [11], distributed across a range of agro-ecological zones including a region of highlands in the central part of the country. Around half of the small ruminant population of Ethiopia are found in this area, mainly in small flocks [12]–[13]. In a recent study, the contact structure of small ruminant flocks in the Bassona Werna *wereda* (region) of the highlands of Ethiopia, based on the use of shared water and grazing points, has been described and analyzed [14]. Using the results of a survey carried out in the same area and within the framework of the same project, the study presented in this paper aimed to investigate the trade patterns among small ruminant traders/farmer traders at village level with the view to set hypotheses on potential factors that explain the observed choices of markets, with special emphasis on the geographical barriers that traders/farmer-traders face at the time to trade livestock. By revealing the underlying structures of a contact network of production units represented by villages in the Bassona Werna *wereda* (Figure 1), these choices might be better understood. Alternative hypotheses about the observed contact structure and the underlying processes that generated it could be also postulated for further studies.

## Methods

### 2.1 Ethics statement

There was not approval obtained by any ethical committee since no human experiments were conducted in this study. The participation to the study required only to answer a questionnaire and participants were assigned a unique code for data entry and analysis remaining anonymous in the further steps of the project. All persons in charge of the field activities of the project were informed and aware of the protocol. Agricultural officers in the area were informed of the nature of the study as well. Consent of participants was obtained orally and in their vernacular language since most of them were illiterate and written consent was not possible. Farmers who were not willing to participate were not forced to. However none of the approached individuals refused to do so. Voluntary participants were informed of the objectives of this study and the data were analyzed and presented in this paper anonymously.

### 2.2 Sampling strategy, data collection and network definition

The highland town of Debre Berhan located in the Bassona Werna *wereda* at 2805 m above the sea level (m.a.s.l.) was used as a base for a set of research activities. The town is located 130 km North along the main road from the capital city of Addis Ababa. Ten out of the 29 *kebelles* of the Bassona Werna *wereda* were preselected for the study according to two accessibility factors: the number of walking days necessary to reach them and the physical ability of the interviewers to reach the *kebelles*. The two most remote *kebelles* were used to pilot the study protocol and questionnaires, with the remaining eight being involved in the main body of research. In each of the eight *kebelles*, 10 villages on average were selected and within each village, 10 small-ruminant owners were selected, using a systematic approach, for individual interviews. Details of the sampling strategy are described elsewhere [14]. During the visits, which took place in February-March 2007, global positioning system location of the village was recorded along with the total number of households and the number of households keeping small ruminants. For each selected individual, the following demographic and trade behaviour data items were collected via a structured questionnaire: name of the trader/farmer-trader, name of the village and *kebelle* of origin, name and frequency of visits to the market for purchase and/or sale during the last year, average number of animals brought to the market, number of sheep and/or goats bought/sold last year, reasons for purchasing livestock, names of the *kebelles* crossed on their way to the market and whether they make a stop and mix with other flocks/herd in the *kebelles* they crossed. Additional attribute data of the villages identified by the interviewees were available from complementary studies in the area [14]. Questionnaire data were used to generate descriptive statistics for variables at village level assumed to reflect the flock contact structure mediated by livestock markets.

A symmetric binary 2-mode network was built linking villages and markets if trader/farmer-trader from a particular village reported to have operated in the market within the time window, i.e., during the year prior to the interview. The two-mode network is a bipartite graph that represents an affiliation network in which nodes of one class, the actors (villages), are linked to nodes of the second class, the events (markets) through the trading choices made by traders. This is so since traders/farmer-traders only traded animals from their villages of origin. The 2-mode network was converted to a 1-mode binary symmetric network of villages linked via trader/s operating in a common market during the time window.

### 2.3.The exponential random graph models (ergm)

A collection of *n* nodes linked via a set of relations (ties, links, edges) constitute a network. In network analysis not only the structure of the relational data can be of interest, but also the attribute characteristics of the nodes and of the edges can be important explanatory variables for the presence of the edge [15]. New developments in statistical network modelling allow researchers to move beyond the mere descriptive approach and test hypotheses about network structure [16]. One of them is a family of statistical models for generalised network inference, the exponential random graph models (ergm), developed as an extension of the first proposed log-linear model for network data: the p^1^ model [17]. The exponential random graph models, referred to as p* models in the social network literature and developed during the 1990 s by Wasserman and Pattison [18] as an extension of the Markov random graph [19], establish a general framework for the estimation of the probability that an edge is present in the network in the logit form, as a linear function of predictors, in a similar fashion as a logistic regression model. The particularity of these models is that the edge appears on both sides of the equation (as outcome and predictor) and often in multiple predictors, making the edge probabilities recursively dependent [20].

In the graph from which the network of this study is derived, the presence or absence of the edge between *n* number of villages (nodes) is defined by an adjacency matrix *Y* of dimension *n* x *n* so that
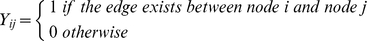



In general, the erg model specifies the probability of a random set *Y* of relations (edges and non-edges) given *y*, a particular set of relations among a set of nodes (villages), namely the observed network, and their attributes, as a function of statistics that may depend on the network itself as well as covariates measured on the nodes , as described by [21]:
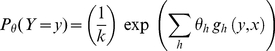
(1)where

*h* is a configuration of the network represented by the observed set of edges among a subset of nodes of the graph containing them; different sets of configuration types represent different models (e.g. dyadic independence or dyadic dependent/Markov random graph) [22];
*g_h_ (y, x)* is a vector of statistics based on the observed adjacency matrix *y*, representing the structure of the network. *x* allows for additional covariate information on the network. The model covariates could include raw network parameters like counts of the configurations in the observed graph (number of reciprocated edges, number of k-stars, number of triangles) but also node or edge-wise covariates like the distance of the village to a certain market or whether the edge is established between villages of the same *kebelle*, respectively. Each covariate should be a function of the observed data. The variables related to the covariates are of the form [23]:
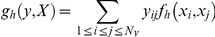

Where *f_h_* is a symmetric function of *x_i_* or *x_j_*, and *x_i_* or *x_j_* are the vector of observed attributes for the *i_th_* or the *j_th_* node. The function *h* can take two forms: an additive one 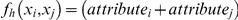 for main effects; and an indicator of the equivalence of the respective attribute of a pair of nodes for second-order effects 
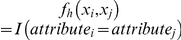

*θ_h_* are non-zero coefficients that denote the statistical parameter governing the probabilistic formation of the network. These are unknown parameters to be estimated.
*k* is a normalization constant and represents the quantity from the numerator summed over all possible networks, so that all probabilities sum to 1.

Eq. [1] can be re-expressed as the conditional log-odds (logit) of individual edges:
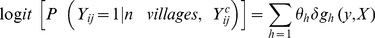
(2)where

*Y^c^_ij_* denotes all edges between nodes *i* and *j* other than the observed *Y_ij_* (the compliment of *Y_ij_* in *Y*), and
*δg_h_(y, X)* is the amount by which *g_h_(y, X)* changes the log odds of an edge when the edge variable *Y_ij_* is changed from 0 to 1 (absence or presence of the edge).

The presence of *Y^c^_ij_* in the conditional probability reflects the mutual dependence of ties. The logit formulation clarifies the interpretation of the *θ* vector: if forming an edge increases *g_h_* by 1, then the log-odds of that edge forming increase by *θ_h_*, with a single edge affecting in some cases multiple *g* statistics [20]. A positive estimate means that the effect is more frequent in the observed network than expected by chance and a negative estimate means that the effect appears less in the network than it could be expected.

### 2.3.1. Dyadic independent erg model

Network inference can be drawn assuming a dyadic independency whereby the state of the dyad (two nodes and their edge) depends on the attributes of the two nodes, for example, but not on the state of other dyads. Under this independency and when fitting these models, the vector of statistics *g_h_ (y, X)* may always be calculated for *Y_ij_*, regardless of the values of *i* and *j*, without knowing anything about *Y*, in the case of an undirected network [24]. Given the difficulty for most networks to calculate the normalizing constant *k*, maximum pseudolikelihood estimation methods (MPLE) have been traditionally applied to estimate the model parameters assuming this conditional independence of the edge (for a review, see Wasserman and Robins [15]), superseded in the last few years by Markov chain Monte Carlo Maximum Likelihood Estimation (MCMCMLE) techniques [25]. Models with only dyadic independent terms have a likelihood function that can be maximized using standard logistic regression methods, as shown above [26].

An initial dyadic independent exponential random graph model was fitted with the edge count as the only non-zero effect in the model, which corresponds with a Bernoulli random graph distribution, often called the simple random graph or Erdös–Renyi graph distribution [22]. To determine the variables in the final model, we used an iterative exploratory technique of progressively decreasing the model complexity by removing variables by decreasing order of p-values from the model containing the edge count and all other pre-selected covariates: pairwise difference in altitude of villages linked in the observed network, euclidean distance to the main market Debre Berhan (measured in decimal degrees), number of small ruminant farmers in the village, number of village sheep sold at the market, number of traders identified during the survey, *kebelle* (first order effect) and *kebelle* (second order effect). The model with the best fit (highest log likelihood) and more parsimonious was selected for reporting and diagnostics. Coefficients and p-values for each covariate and log likelihood and the Akaike Information Criterion (AIC) for the final model were extracted and displayed in Table 1.

**Table 1:** Descriptive statistics of the village attributes, coefficients and p values, AIC and log likelihood parameters of the three erg models: the edge count and of the final dyadic independent and dependent models.

Covariates	Descriptive statistics Median IQR (25^th^–75^th^)	Edge	Erg dyadic independent model Coefficients(P value)	Erg dyadic dependent model Coefficients(P value)
Edge		**−0.32 (<0.05)**	**−9.64 (<0.05)**	**−112.3 (<0.05)**
Absolute difference altitude (m.a.s.l.)	3008.5 (2813–3116)		**0.003 (<0.05)**	
Distance to Debre Aber (decimal degrees)	0.13 (0.11–0.16)		**29.9 (<0.05)**	
Number of small ruminant farmers	27 (21–34)			
Total number of sheep sold at the market	16 (12–29)			
Number of traders identified during the survey	10 (10–10)			
Kebelle (second order effect):				
Abamote			**−1.3 (<0.05)**	
Angolela			0.6 (0.07)	
Bere Ager			0.6 (0.07)	
Birbisa			−1 (0.08)	
Debele			−0.1 (0.73)	
Goshebado			-Inf (NA)	
Gudoberet			**−1.5 (<0.05)**	
Keyit			0.08 (0.8)	
2-star				**2 (<0.05)**
3-star				**−0.01 (<0.05)**
Triangle				**−1.7 (<0.05)**
Log-likelihood		−1888.3	−1418.87	−46.9
AIC		3778.6	2859.7	101.8

### 2.3.2 Dyadic dependent erg model

When the likelihood of a dyad depends on the presence or the state of other dyads, the models to account for this dependency require computationally intensive estimation and imply complex forms of feedback and global dependence that “confound both intuition and estimation” [26]. The fitting of these models are based on an algorithm that draws on Markov Chain Monte Carlo simulations (MCMC), a stochastic process that produce different results every time they are run, unlike dyadic independent models.

**Figure 2:**
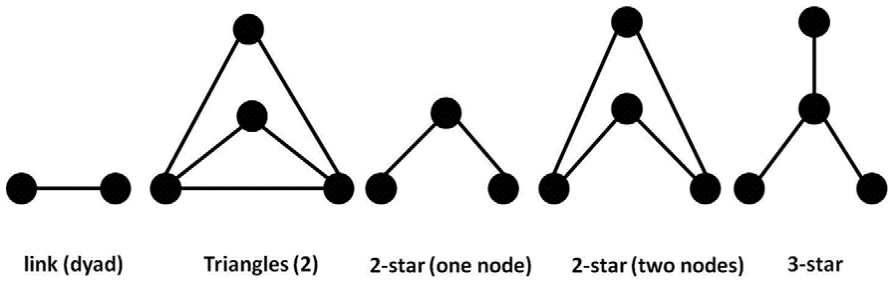
Examples of the network configurations included in the dyadic dependent exponential random graph model.

In order to describe the internal structure of the study network a dyadic dependent exponential random graph model was fitted using some of the traditional configurations in undirected networks that describe the structural cohesiveness of the network: the k-star (2-star and 3-star) and the triangle, apart from the edge count, as in the previous model. A 2-star is a subset of three nodes in which one node is connected to each of the other two, and a triangle is a subset of three mutually connected nodes. These configurations are defined hierarchically, so that a triangle also includes three 2-stars [22]. The statistics estimated in the model are related to the count of these structures presented in the observed network (Figure 2). To prevent the degeneracy of the model the MCMC sample size was increased up to 100,000 [27].

### 2.3.3 Goodness-of-fit test and model diagnostics

In order to check if the selected final models capture the structure of the original observed network, a set of 100 randomly generated networks were simulated using the parameters of the fitted final model. They were then compared with the observed network by four diagnostic parameters as proposed by Hunter et al. [24]:

geodesic distance distribution defined as the proportion of pairs of nodes whose shortest connecting path is of length k, for k = 1, 2…*m* (pairs of nodes that are not connected are classified as k = 1);
the edgewise shared partner distribution: based on the number of edges that serve as the common base for exactly *i* distinct triangles, expressing the tendency in the observed network for linked nodes to have multiple shared partners [21];
the degree distribution or the frequency of nodes with different degree values;
and the triad census distribution defined as the proportion of 3-node sets having 0, 1, 2, or 3 edges among them.

Frequency distributions of the four diagnostic parameters were produced for the observed data (the study network) and the 100 simulated networks. This was conducted using the built-in goodness of fit method in the package *statnet* of the statistical software R [28]. For good-fitting models, the plot of the simulated networks should closely match that of the observed network.

The statistical estimates of the parameters of the erg models indicate whether network realizations with the theoretically hypothesized properties have significantly large probabilities of being observed in subgraphs of the network data collected. Following this rationale, a further diagnostic of the final models was conducted by the following procedure: firstly, one edge of the original network was removed. Then a set of 100 randomly generated networks were simulated using the covariates of the final models fitted with new network (the original minus one edge). The number of times that the eliminated edge is included in the simulated networks was counted. This procedure was repeated by selecting randomly 10 edges present in the network and removing one at a time. Average number of times the edges are included in their respective simulated batch of one hundred networks is reported, as a measure of the reproducibility of the edge present in the observed network and subsequently removed using the covariates of the final models.

For the dyadic dependent model and in order to test its degeneracy, plots of the chain for each model statistic produced in every MCMC sample were produced. Visual exploration was conducted to check whether the statistics of the model vary stochastically around the mean as expected in a converged model and do not depart steadily away from the mean [27]. All the analyses were conducted using the statistical software R version 2.12.0 (R Development Core Team (2010). R: A language and environment for statistical computing. R Foundation for Statistical Computing, Vienna, Austria. ISBN 3-900051-07-0, URL http://www.R-project.org).

## Results

841 responses were collected with information on either the trader or the market or both. Seventy seven responses did not include the name of the trader or farmer-trader. Of those with names, five individuals reported two markets each where they had traded and 759 only declared a single market, making a total of 764 different individuals reporting trade in the survey.

A hundred and ninety two individuals did not provide information on the number of animals traded but they provided the markets where they traded. Two did not specify market name, 194 mentioned market “none”, 4 mentioned “village” as the market where they traded, making a total of 9 markets identified and 570 observations where a different pair of trader/farmer-trader and market/s could be both identified and were included in the final dataset for analysis. The 9 markets identified were: Abadale, Ankober, Arbgebeya, Chacha, Debre Berhan, Gudoberet, Keyit, Mendida and Rob gebeya. A total of 75 different villages from the 8 *kebelles* were identified in the 570 paired observations. Locations of both the villages and markets included in the analysis are shown in Figure 1.

The median number of visits to the market by traders/farmer-traders during the time window was 2 (IQR: 1–2, range 1–52). Among those who sold sheep in the markets (569), the median number of sheep sold was 2 (IQR = 1–3, range: 1–32). The most frequent reason for selling sheep was ‘to buy clothes’ (59%), followed by ‘to buy food’ (43%), ‘to buy fertilizer’ (35%), ‘to pay taxes’ (29%), ‘to pay school fees’ (23%) and ‘to buy feed for animals’ (14%). In terms of priority, these were also the reasons to sell in decreasing order of priority for the 528 respondents to this question. Among those who sold goats (50), the median number of sheep sold was 1 (IQR = 1–2, range: 1–8). The traders/farmer-traders included in the survey were mainly suppliers and only 97 (17%) of them reported to have bought sheep during the reporting period (median: 2, IQR:1–2, range: 1–10). Even less individuals purchased goats (7), mostly single animals. The main reasons to buy sheep were: ‘for own consumption’ (50%), ‘for breeding’ (40%) and ‘for fattening and sale’ (10%).

More than a third of the 563 respondents to this question did not cross any other *kebelle* on their way to the market (39%) and when they do it, they stopped at other *kebelles* in 85% of the occasions and usually mixing with other herds 9 out of 10 times. Only 7 respondents crossed three *kebelles* (1%).

The 1-mode network contained 75 villages from 8 different *kebelles* in the Bassona Werna *wereda*. It is a dense network (42%) with a median degree of 42 (IQR: 15–53), an overall clustering coefficient of 0.37 and average geodesic distance of 1.5. These features are due to the dominant effect of the main market Debre Berhan in which traders from 54 villages (72%) operated during the reporting period. Descriptive statistics of the main village attributes are displayed in Table 1.

The negative coefficient in the initial model including only the edge count indicates fewer connections between villages in the network than would have been expected by chance (p<0.05).

The final dyadic independent erg model included the edge count, pairwise difference in altitude of the villages linked, distance to the main market of Debre Berhan and the second order effect of the *kebelle*, all significant at the 0.05 level. The odds of a edge increases significantly where the distance to the market Debre Behran and the difference in altitude between two connected villages are both large so that villages far away from the main market and at different altitude are more likely to be linked in the network than randomly. The odds of forming an edge between two villages in Abamote or Gudoberet adjusted by distance to Debre Berhan and altitude are approximately 75% lower than an edge between villages in any other *kebelles* (p<0.05 for both villages), conditional on the rest of the network, whereas edges between villages in Angolela and Bere Ager were more likely to occur than randomly and less likely between villages in Birbisa, although only significant at the alpha level of 0.1.

The final dyadic dependent erg model included the counts of edges, 2-star, 3-star and triangles configurations. Following the interpretation of the coefficients, the conditional log-odds of two villages forming a tie that is not included in a triangle, a 2-star or a 3-star is extremely low as the large coefficient of the edge count shows, increasing the odds significantly (p<0.05) each time the node is in one 2-star structure and decreasing it when a node is in a 3-star (p<0.05) or in a triangle formation (p<0.05). Parameters estimates and p-values as well as log likelihood and AIC of the three models are shown in Table 1.

**Figure 3:**
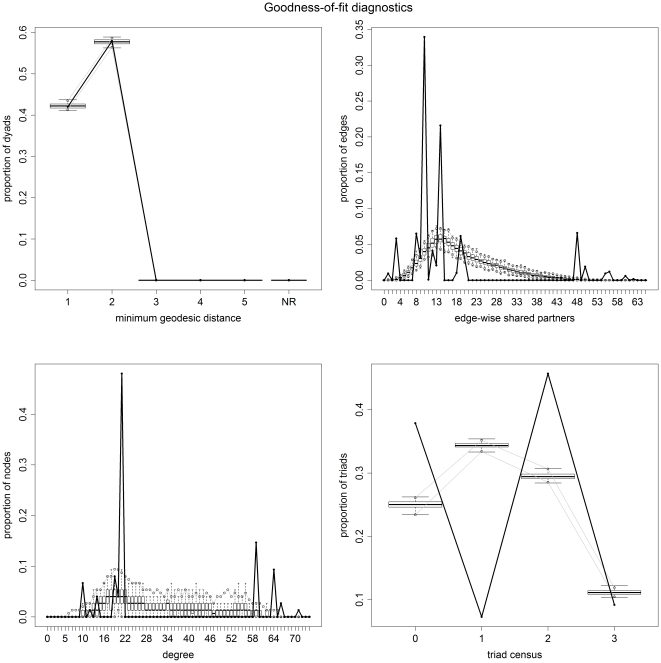
Plots of the proportion of dyads against the four diagnostic parameters of both the observed networks (black) and the 100 simulated networks (grey) for the dyadic independent model. The solid lines represent the statistics of the observed network, and the boxplots represent the distribution of 100 simulated networks based on the fitted ergm.

**Figure 4:**
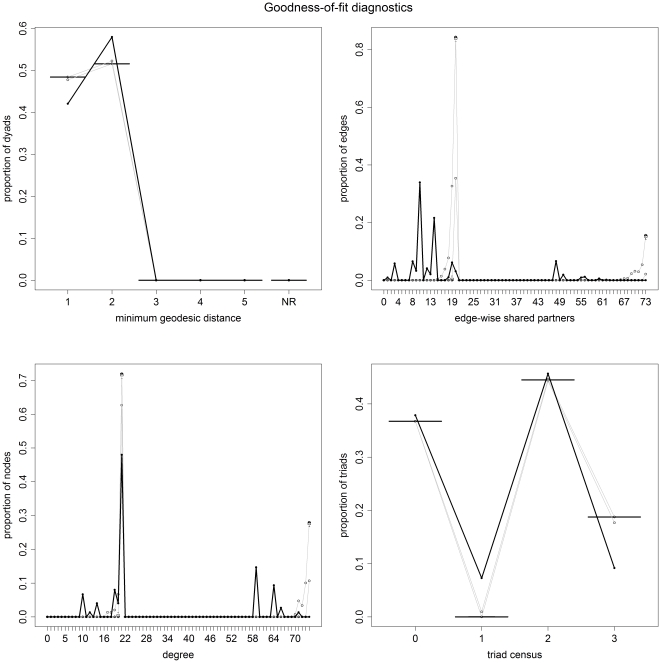
Plots of the proportion of dyads against the four diagnostic parameters of both the observed networks (black) and the 100 simulated networks (grey) for the dyadic dependent model. The solid lines represent the statistics of the observed network, and the boxplots represent the distribution of 100 simulated networks based on the fitted ergm.

**Figure 5:**
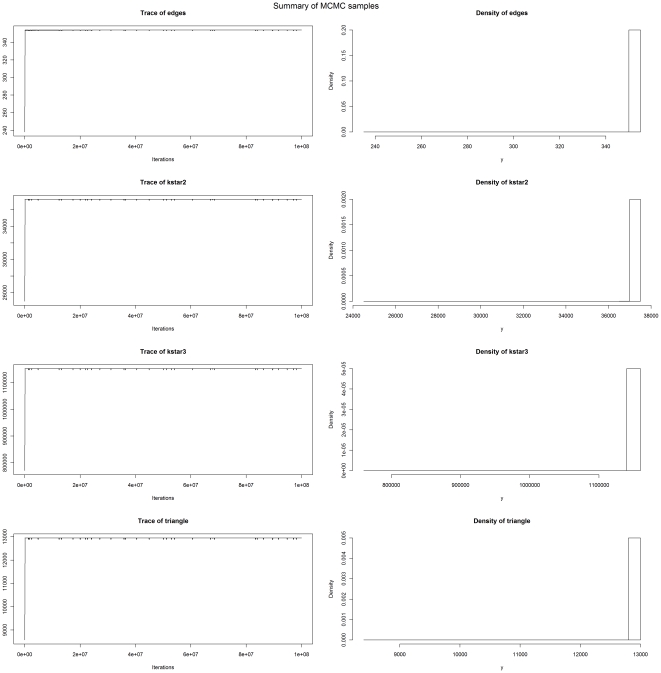
Plot of the statistics estimated in each MCMC sample for the dyadic dependent (left) and the frequency histogram of the estimation of the parameters of the model (right) using a MCMC sample size of 100000.

The frequency distributions of the four diagnostic parameters of both the observed network and the 100 simulated networks for the dyadic independent and dyadic dependent models are displayed in Figure 3 and Figure 4, respectively. The independent or attribute-related model does a good job in capturing the global efficiency of the network (geodesic distances), a relative good fitting for 2 and 3 triad census, but predicts poorly the local efficiency (edge-wise shared partners) and the degree distribution due to the bimodal distribution of degree in the network whereby nodes have degree below 15 or over 50. On the other side the dyadic dependent model is able to replicate the four diagnostic parameters of the observed network much more accurately, with some predicted outliers of nodes with low edge-wise shared partners. The plots of the statistics estimated in each MCMC sample of the parameters of the dyadic dependent model are shown in Figure 5. Visually the model appears to converge with no deviation of the parameter estimators from the mean values.

The randomly removed edge appeared on average in 14% of the simulated networks for each batch in the dyadic independent model and in 15.5% in the dyadic dependent model.

## Discussion

The highland town of Debre Berhan (elevation 2805 m.a.s.l.) located in the Bassona Werna *wereda* was used as a base for the research. The town is located 130 km North along the main road from the capital city of Addis Ababa. The market system observed in this community of small ruminants of Ethiopia is dominated by a large market, Debre Berhan, that serves as a meeting point for farmer/farmer-traders to buy/sell small batches of mainly sheep and at a smaller scale goats. It lays on Road 1, one of the main arteries of the road network in Ethiopia, stretching from Addis Ababa to the border with Eritrea in the north.

Following qualitative data collected during the survey and the parameters of the observed network, most of the markets studied are medium/small scale located far from the main road, and play a secondary role in bringing in a few animals at a time which are sold onto farmer-traders or small-scale traders and then moved to other markets like Debre Berhan. These however, are often accessible by car on dirt or main roads. This centripetal general flow of live animals from production sites to larger towns is characteristic of the supply chain of livestock production in this setting [6]–[29]–[30]. The dynamism and complexity of the system reflects the opportunities to make a profit by trading with small ruminants. Traders' strategies include attending several markets each week and following a gradient of prices from the more isolated locations to larger towns and/or the capital.

It is extremely difficult to collect reliable field data on this type of settings where trade information is the main objective of the questionnaires. Not only because the unfamiliarity of the subjects to this kind of studies but also the lack of standard denomination for villages and markets alike. Despite these downsides of the data collected in this study, the analysis revealed certain patterns in the contact of production units represented by villages through the trade of small ruminants via markets.

The exponential random graph models provide a statistical framework to analyse network data by modelling the probability that any given graph is drawn from the same distribution as the observed graph. They also allow different network structures to be modelled, because the formulation is able to account for the complex structure of the network via parameters governing the entire network, rather than breaking it down into dyads [31]. Two different outputs can be extracted by fitting an erg model: the prediction of the probability of the observed overall network structure and/or the likelihood of any specific edge in an observed network. Another advantage is that the outputs of these models are interpreted in a similar manner as standard logistic regression.

The dyadic independent village-village network model shows dyadic independence because the probability of any edge does not depend on the value of or the presence of other edges, only on the attributes of the two villages (node) involved in the edge [20]. The similarity effect is strong with the distance to the main market and the difference in altitude. The edge parameter is increased/decreased to compensate the effect of the other covariates from the initial model that only contain it. This is an indicator of the density or overall cohesiveness of the network. Reading the results we conclude that there were fewer edges in this network than expected, that is, many fewer dyads of villages linked via common markets that had no other ties. In the context of the study two major constraints could be expected to influence traders/framer traders on which market to attend: distance and geographical barriers expressed in our dataset by the euclidean distance to the main market measured in decimal degrees and difference in altitude from the low land to higher, respectively. Although small in the log of the odds of the edge, the difference in altitude is higher in the network than expected and so is the distance to the main market of Debre Berhan at larger scale, which means that the geographical barriers and distance do not determine the decision on which market to trade and hence to be linked to other village of the study network. If the pattern observed was extrapolated to a larger population of small ruminant farmers, the catchment area of a market could not be estimated based on distance but on other criteria like type of market, price differential and opportunities for social interaction. In this regard Debre Berhan is on major road and the advantages of taking sheep and goats to this main market may outpower the difficulties of moving animals longer distances, from lower areas and crossing other villages contacting other flocks. The drawback of this fact is the opportunities for mixing in the way to the market.

The first model based on the attribute-related dyadic independency also showed the assortative mixing of villages by *kebelle* whereby villages within two *kebelles*, Abamote and Gudoberet, are linked less frequently than expected adjusted by distance to the main market and the difference in altitude. Although the overall effect of the network model reveals that the difference in altitude of two villages does not preclude to be linked, a potential explanation for this finding is the fact that these two *kebelles* are located in the remotest region of the *wereda* and they may tend to trade via small local markets reducing their opportunities to be linked via the larger markets identified in the study. Other attributes inherent to the 8 *kebelles* identified in the network and unaccounted for in this analysis may explain this assortative mixing.

The second model contained the dyadic dependency leading to an endogenous process of formation of ties in the form of internal structures (stars, triangles, etc.). Yet again the negative density parameter indicates that edges occur very rarely (large negative coefficient), especially if they are not part of higher order structures such as stars and triangles. The negative triangle parameter can be interpreted as providing evidence that the edge between villages do not tend to occur in triangular structures, and hence cluster into clique-like forms. The transitive triangle parameter is an indicator of clustering and strength [32]. This statistic is interpreted as the tendency for many triangles to form together in the observed network. If high, then the model suggests regions of high triangulation indicative of core-periphery-type structures [33].

The star effects are significant suggesting that there is a tendency for multiple network partners up to degree of 2 (the positive 2-star estimate) but with a ceiling on this tendency (the negative 3-star parameter), both significant. *k*-stars are equivalent to geometrically weighted degree counts and are useful for modelling the degree distribution. In fact 1-star is equivalent to the degree of the nodes. The higher the *k*-star parameter, the easier it is for information/commodities to circulate through the network [32]. In this regard the structure of the study network showed some resilience to spread diseases globally assuming that the causative agent is mobilized via movement of small ruminants in the network.

Both models have a low reproducibility of individual edges with 14% in the attribute-related model and 15.5% in the configuration-related model. Internal structures in the network allow a better prediction of individual edges than the attributes of the node, although with a small advantage. However the dyadic dependent model predicts much better the overall structure of the network according to the four diagnostic parameters and the log likelihood of the model.

The results of the study preclude the effect of geographical barriers on the choices that traders/farmer-traders make to trade small ruminants in the study area. It could have been expected the environment to play a role in “constraining” disease transmission routes by the physical impediment of bringing animals into contact in the setting of the study. However it has been shown that the two major constraining factors, namely distance and altitude, are not deterrent for the potential contact of susceptible small ruminant populations in the Highlands of Ethiopia. It has also been observed the assortative mixing of the villages via common markets by *kebelles*. The attribute data collected at village level and included in the analysis captured a limited variability of the probability of the presence of the edge and other factors unaccounted for would definitely complement the trading criteria of the traders/farmer-traders to make their choices.
